# Identification and Functional Characterization of a Novel Mutation in the Human Calcium-Sensing Receptor That Co-Segregates With Autosomal-Dominant Hypocalcemia

**DOI:** 10.3389/fendo.2018.00200

**Published:** 2018-04-25

**Authors:** Anne Qvist Rasmussen, Niklas Rye Jørgensen, Peter Schwarz

**Affiliations:** ^1^Research Centre of Ageing and Osteoporosis, Department of Endocrinology, Rigshospitalet, Copenhagen, Denmark; ^2^Department of Clinical Biochemistry, Rigshospitalet, Copenhagen, Denmark; ^3^Institute of Clinical Research, University of Southern, Odense, Denmark; ^4^Faculty of Health Sciences, Copenhagen University, Copenhagen, Denmark

**Keywords:** calcium-sensing receptor, “gain-of-function” mutation, GPCR, signaling, ADH

## Abstract

The human calcium-sensing receptor (*CASR*) is the key controller of extracellular Ca_o_^2+^ homeostasis, and different mutations in the *CASR* gene have been linked to different calcium diseases, such as familial hypocalciuric hypercalcemia, severe hyperparathyroidism, autosomal-dominant hypocalcemia (ADH), and Bartter’s syndrome type V. In this study, two generations of a family with biochemically and clinically confirmed ADH who suffered severe muscle pain, arthralgia, tetany, abdominal pain, and fatigue were evaluated for mutations in the *CASR* gene. The study comprises genotyping of all family members, functional characterization of a potential mutant receptor by *in vitro* analysis related to the wild-type receptor to reveal an association between the genotype and phenotype in the affected family members. The *in vitro* analysis of functional characteristics includes measurements of inositol trisphosphate accumulation, Ca^2+^ mobilization in response to [Ca^2+^]_o_-stimulation and receptor expression. The results reveal a significant leftward shift of inositol trisphosphate accumulation as a result of the “gain-of-function” mutant receptor and surprisingly a normalization of the response in (Ca^2+^)_i_ release in the downstream pathway and additionally the maximal response of (Ca^2+^)_i_ release was significantly decreased compared to the wild type. However, no gross differences were seen in D126V and the D126V/WT *CASR* dimeric >250 kDa band expression compared to the WT receptor, however, the D126V and D126V/WT CASR immature ~140 kDa species appear to have reduced expression compared to the WT receptor. In conclusion, in this study, a family with a clinical diagnosis of ADH in two generations was evaluated to identify a mutation in the *CASR* gene and reveal an association between genotype and phenotype in the affected family members. The clinical condition was caused by a novel, activating, missense mutation (D126V) in the *CASR* gene and the *in vitro* functional characteristics of the mutation co-segregated with their individual phenotype.

## Introduction

In humans, extracellular calcium concentrations ((Ca^2+^)_o_) play a key role in most vital physiological processes, and thus a tight control of (Ca^2+^)_o_ is critically important. The G protein-coupled calcium-sensing receptor (*CASR*) plays a major role in systemic calcium homeostasis. The most important physiological function of *CASR* is to maintain and regulate (Ca^2+^)_o_. In humans, *CASR* is highly expressed on the cell membrane of the chief cells of the parathyroid glands, where it "senses" the change in (Ca^2+^)_o_ and subsequently generates and alters the release of parathyroid hormone (PTH) (1, 2). Moreover, *CASR* is functionally expressed in the bone, kidney, and gut, the three major organs involved in calcium homeostasis. (Ca^2+^)_o_ has key roles in bone biology, in the regulation of different hormones, and in various cellular functions. *CASR* is the first known receptor that permits an ion (Ca^2+^) to have a “hormone like” role as a first messenger in addition to its well-known role as an intracellular second messenger (3–6). In the kidney, *CASR* plays a pivotal role in renal calcium reabsorption and excretion (7), whereas it regulates calcium absorption in the intestine (8, 9). In bone, *CASR* controls the mineralization of the matrix and regulates bone turnover (10). The blood ionized calcium concentration results from the balance between intestinal calcium absorption, renal calcium reabsorption, and/or calcium release from bones. In humans, *CASR* responds to even very small fluctuations in the (Ca^2+^)_o_, and PTH acts within seconds or minutes to reestablish the (Ca^2+^)_o_ in humans, showing a very steep inverse sigmoidal relationship between (Ca^2+^)_o_ and PTH (1, 2, 11). The calcium set-point has been used for the clinical evaluation of calcium-related diseases (12–14). If the regulation of (Ca^2+^)_o_ fails, several vital organs will be affected. Most importantly, hypocalcemia will induce increased excitability of the nervous system with a risk of tetany, which occurs when (Ca^2+^)_o_ is reduced by 0.40 mmol/L below baseline (15).

The receptor is activated when endogenous ligands bind to one of the three binding sites in the large N-terminal “Venus FlyTrap” domain and/or to the one in the cys-rich domain, respectively (16). Consequently, changing the configuration of the receptor and subsequently the downstream signaling and interaction with the associated G-protein. The primary endogenous ligand of *CASR* is (Ca^2+^)_o_, although *CASR* also responds to other inorganic di- and trivalent cations, as well as naturally occurring l-amino acids (17). The principal signaling pathway of *CASR* is the Gα_q/11_-activated phospholipase C_β_ pathway (PI-PLC). (Ca^2+^)_o_ binding to dimeric *CASR* activates the receptor, mediates the accumulation of inositol trisphosphate (IP), and subsequently increases the intracellular Ca^2+^ concentrations ((Ca^2+^)_i_) *via* mobilization from intracellular stores (18, 19). In human parathyroid cells, the increase in (Ca^2+^)_i_, together with vitamin D response element, inhibits the transcription of the gene encoding PTH (20, 21), generating an increase in renal Ca^2+^ excretion and subsequent a decrease in intestinal Ca^2+^ absorption, which results in a decrease in (Ca^2+^)_o_ (22, 23).

A number of mutations in the *CASR* gene have been shown to affect calcium homeostasis by changing the calcium set point, PTH secretion, bone resorption, and renal calcium excretion. Clinically, mutations in *CASR* can either cause hypercalcemia if the receptor is inactivated (loss-of-function), resulting in familial hypocalciuric hypercalcemia, or hypocalcemia if the receptor is activated (gain-of-function) to produce a hypersensitive receptor, causing autosomal-dominant hypocalcemia (ADH) (13). Recent studies have shown that the pleiotropic *CASR* gene induces agonist- and ligand-biased signaling *via* pathways coupled to the PI-PLC, p38 MAPK, and JNK pathways (24–27), indicating that the signaling bias is an important property of the receptor function that might explain the key differences in receptor function between tissues and the patterns of diseases arising from specific disease-related mutations. We report here a novel “gain-of-function” mutation of the *CASR* that co-segregated with the individual phenotype in two generations diagnosed ADH. The results indicate that the naturally occurring mutation in the receptor may have engendered a change in receptor conformation that biases the signaling.

## Subjects and Methods

### Patients

In this study, two generations with biochemically and clinical confirmed ADH who suffered from severe muscle pain, arthralgia, tetany, abdominal pain, and fatigue were evaluated for mutations in the *CASR* gene. The proband, individual I-2, was a 42-year-old woman who suffered from chronic hypocalcemia, i.e., a serum ionized calcium concentration of <1.15 mmol/L, with no increase in serum PTH levels, normal kidney function, low serum 25-hydroxyvitamin D levels, and normal 1,25-dihydroxyvitamin D levels (Table 1). Her baseline values were further controlled to exclude Bartter’s syndrome and she had a S-sodium of 143 mmol/L, S-potassium 3.9 mmol/L, S-creatinine of 89 mmol/L, S-magnesium of 0.65 mmol/L, and the U-Ca/Crea was 0.12 (reference < 0.14).

**Table 1:** The biochemical baseline characteristics of the ADH family members investigated.

Patient	Age (years)	BMI	BMD_(L2–L4)_ (g/cm^2^)	S-Ca_(total)_ (mmol/L)	S-Ca^2**+**^ (mmol/L)	S-PTH(1–84) (pmol/L)	S-PO^4**−**^ (mmol/L)	S-Mg^2**+**^ (mmol/L)	S-1,25(OH)_2_D (pmol/L)	S-25(OH)D (nmol/L)	U-Ca (mmol/L)
Ref. range DS/EN ISO 15189:2013				2.20–2.60	1.15–1.35	1.1–6.9	0.80–1.50	0.67–0.93	51–177	50–160	2.00–7.00
Ι-1_♂_	47	NA	NA	NA	1.21	6.9	NA	NA	NA	68	NA
Ι-2_♀_	42	24.1	1.107	**1.90**	**1.02**	**<0.6**	1.0	**0.65**	130	**26**	4.7
ΙΙ-1_♂_	19	22.4	0.580	2.48	1.26	1.9	NA	NA	NA	**24**	5.9
ΙΙ-2_♂_	17	19.8	0.914	**2.08**	**1.06**	**0.9**	0.99	0.78	99	**45**	6.5
ΙΙ-3_♀_	16	17.9	1.084	**1.89**	**1.02**	1.3	**1.54**	0.73	113	56	5.2

*Data in bold are out of reference range*.

*NA, not analyzed; ADH, autosomal-dominant hypocalcemia; PTH, parathyroid hormone; BMD, bone mineral density*.

One child (II-3) showed clinical symptoms that were similar to the proband. Family members II-2 and II-1 reported some clinically insignificant symptoms of skeletal and muscle pain. Dual-energy X-ray absorptiometry-scanning of the spine and hip showed that family member II-2 (17 years, male, height 180 cm, weight 64.0 kg) was osteopenic, with a bone mineral density (BMD) of 0.914 g/cm^2^ at the lumbar spine (L_2_–L_4_). Family member II-1 (19 years, male, height 187 cm, weight 78.2 kg) had a low BMD of 0.580 g/cm^2^ at the lumbar spine (L_2_–L_4_). The proband (height 169 cm, weight 68.9 kg) and II-3 (II-3, 16 years, female, height 164 cm, weight 48.1 kg) both had normal lumbar spine BMD values of 1.107 and 1.084 g/cm^2^, respectively. The proband and the II-3 showed micro calcifications in various tissues in addition to nephrocalcinosis. In addition, the proband had Quincke’s edema, symptoms of urticaria, decreased muscle strength, and infrequent muscle cramps in the extremities, whereas II-3 showed additional frequent depressive periods. The baseline biochemical characteristics are presented in Table 1. An evaluation of urinary calcium excretion showed that the levels were between 5 and 9 mM/day in all affected family members. Peripheral blood was collected by venipuncture from the proband, her husband, and their three children to genotype the *CASR* mutations. The participants gave written consent for use of biological material for scientific purposes. The study was approved by the Unit of Health Care, Regional Scientific Ethical Committee, The Capital Region of Denmark (J.no.17011317).

### Methods

#### Sequence Analysis

DNA was purified from peripheral blood and the sequences of the UTR exon 1A/B and the protein-coding exons 2–7 of the *CASR* gene, including the related boundaries, were analyzed after designing primers for all 9 exons used for PCR amplification, in addition to direct sequence analysis of the PCR amplicons (see the nucleotide sequences for the primers in Table S1 in Supplementary Material). The DNA sequences were amplified using standard PCR techniques as follows: 1 cycle of denaturation at 94°C for 10 min, then 30 cycles of denaturation at 94°C for 30 s, hybridization of the designed primers at 60°C for 30 s, except for the primers for exon 4 which were hybridized at 62°C for 30 s, and subsequent elongation at 72°C in 30 s, followed by a final elongation at 72°C for 10 min. Ampli *Taq-*gold DNA polymerase (Applied Biosystems, Foster City, CA, USA) was used to amplify all exons. The PCR amplicons were verified by gel electrophoresis. Subsequently, the nucleotide sequence of both strands was determined by direct sequencing using the ABI Prism^®^ BigDye™ Terminator, which was analyzed on an Applied Biosystems 3100 Genetic Analyzer (ABI3100), according to the manufacturers’ instructions. The nucleotide sequence identified by the ABI3100 was analyzed with the SeqScape Software ver. 2.0 (Applied Biosystems) to confirm any variations, single-nucleotide polymorphisms or mutations using the reference sequence of the *CASR* gene (NG_009058).

#### Construction of the Wild-Type and Mutant *CASR* Vector

The wild-type calcium-sensing receptor vector (WT-*CASR*) is a construct of the Human Pearce cassette WT-*CASR* cDNA cloned into the pcDNA3.1(+)vector (Invitrogen™, Thermo Fisher Scientific Inc., MA, USA) using the *Kpn* I and *Xba* I restriction sites in the multiple cloning site of the pcDNA3.1(+)vector and was termed rHuPCAR4.0 (28). The rHuPCAR4.0 vector was supplied by A.D. Conigrave, School of Molecular and Microbial Biosciences, University of Sydney, NSW, Australia, and originated from M. Bai and E. M. Brown, Division of Endocrinology, Diabetes, and Hypertension, Department of Medicine, Brigham and Women’s Hospital, Boston, MA, USA. Site-directed mutagenesis was performed on the human WT-*CASR* cDNA pc3.1DNA(+)vector to produce the specific missense mutation corresponding to substitution of an adenosine nucleotide (A) for thymidine (T) in codon 126 (GAT) of the *CASR* gene, which causes a change from the amino acid aspartic acid to valine in the receptor protein. The site-directed mutagenesis and subsequent control sequencing were performed by Eurofins Genomic, Ebersberg, Germany.

#### Cell Culture

Flp-In human embryonic kidney-293 (HEK293) cells were cultured in Dulbecco’s Modified Eagle’s Medium (DMEM) with 4.5 g/L glucose and l-glutamine (Lonza #614712F, Basel, Switzerland) supplemented with 10% fetal calf serum (FCS), 0.5 U/mL penicillin, 0.5 mg/mL streptomycin, and zeocin 0.1 mg/mL (Invitrogen™). The cells were maintained in a T-175 cell flask and incubated at 37°C in a humidified 5% CO_2_ atmosphere. The Flp-In HEK293 cells were a gift from Hans Braüner-Osborne, Department of Drug Design and Pharmacology, Faculty of Health and Medicine Science, University of Copenhagen, Denmark.

#### Transfection of Cells

The plasmids encoding the mutant and WT receptors were transiently transfected into Flp-In HEK293 cells to study the functional characteristics of the mutant *CASR* protein *in vitro*. One day before the transient transfection of the cells for the functional assays, 2 × 10^4^ cells per well were seeded into a clear 96-well plate for the IP assay and into a black, clear bottom plate for the (Ca^2+^)_i_ mobilization assay. For the Western blot experiments, 1 × 10^6^ cells per well were seeded in 6-well plates. The plates were coated with poly-d-lysine (50 µg/mL) to allow for sufficient cell adhesion. On the following day, the cells were transfected with either 0.25 μg/well of the WT-*CASR*-pcDNA3.1(+), mutant-*CASR*-pcDNA3.1(+), WT- and mutant-*CASR*-pcDNA3.1(+), or mock-vector using Lipofectamine (Invitrogen™) in a 1:1 ratio for the functional assays. However, for Western blotting, a 1:2.5 ratio was transfected according to the manufacturer’s protocol. The cells were maintained 16–24 h in DMEM with 4.5 g/L glucose and l-glutamine (Lonza # 614712 F) and 10% FCS. The cells were incubated at 37°C in a humidified 5% CO_2_ atmosphere.

#### IP Accumulation Assay

*In vitro* functional characterization of the individual receptor proteins was performed within 48 h of the transfection. The cells were analyzed for accumulation of soluble inositol phosphate (IP) in the presence of lithium, using a phosphatidylinositol hydrolysis assay as previously described (29). In brief, the day after transfection, the cells were washed twice with assay buffer (HBSS with calcium and magnesium) (Life Technologies # 14025, Thermo Fisher Scientific Inc., MA, USA), supplemented with 20 mM HEPES (Life Technologies # 15630-056, Thermo Fisher Scientific Inc., MA, USA, at pH 7.4), and incubated 16–24 h with 100 µl of inositol-free DMEM (MP Biomedical, Santa Ana, CA, USA cat # 1642954) supplemented with 10% FCS and 1 μCi/well myo-[2-^3^H] Inositol (code TRK 911, GE Healthcare, Little Chalfont, UK). After incubation, the medium was removed; the cells were washed twice with assay buffer, and then incubated with 100 µl of assay buffer supplemented with 10 mM LiCl preventing dephosphorylation of the accumulated IP by inositol phosphatase for 20 min at 37°C. The buffer was removed, and the cells were incubated with 100 µL of HBSS buffer without calcium and magnesium supplemented with 10 mM LiCl and various concentrations of CaCl_2_ for 60 min. The reactions were stopped by exchanging the buffer with 50 µL of ice-cold 20 mM formic acid and incubated for 1 h on ice to release the IP generated by the cells. Thirty microliters of the lysis solution were transferred to a solid white flat bottom 96-well plate (NUNC, Thermo Fisher Scientific Inc., MA, USA) and 1 mg/mL SPA-YSi scintillation beads (PerkinElmer, Boston, MA, USA) was added to each well. The plate was sealed, shaken for 1 h, and centrifuged at 1,500 rpm for 5 min. The labeled IP was measured by detecting the emitted light using a MicroBeta^2^ Plate Counter (PerkinElmer).

#### (Ca^2+^)_i_ Mobilization Assay

*In vitro* functional characterization of the individual receptor proteins was performed within 48 h of the transfection. The cells were analyzed for the release of (Ca^2+^)_i_ from the endoplasmic reticulum, as previously described (30). In brief, the medium was removed from the cells, 50 µl of assay buffer (HBSS w/o calcium and magnesium) (Life Technologies # 14175) supplemented with 1 mM CaCl_2_, 1 mM MgCl_2_, 20 mM HEPES, 2.5 mM probenecid, and 4 µM fluo-4AM (acetoxymethyl ester) (Invitrogen™), pH 7.4, were added, and the cells incubated for 1 h at 37°C in a humidified 5% CO_2_ atmosphere. The cells were washed with wash buffer (assay buffer without fluo-4AM) and were placed in the incubator for 30 min after the addition of 100 µl of fresh wash buffer. (Ca^2+^)_i_ release was quantified by measuring the fluorescence at 485 nm excitation and 525 nm emission on a NOVOstar (Molecular Devices; Sunnyvale, Ca, USA) for 60 s after the addition of various concentrations of CaCl_2_. Changes in the relative fluorescence units [ΔRFU = (*F* − *F*_0_)/*F*_0_] of Fluo-4 AM-loaded Flp-In HEK293 cells were calculated to adjust for the differences in cell number and loading efficiency (*F* expresses the maximal response of fluorescence, *F*_0_ expresses the basal response of fluorescence.).

#### Immunoblotting

*In vitro* functional characterization of the individual receptor proteins was performed within 48 h of the transfection. The cells were washed once with ice-cold phosphate-buffered saline. Afterward, the cells were lysed in ice-cold lysis-buffer (10 mM Tris–HCl, pH 7.4, 10 mM NaCl_2_, 3 mMgCl_2_, and 1 mM EDTA) containing 3% NP-40 detergent and 12.5% protease inhibitors (Complete tablets, ref # 04693116001, Hoffmann-La-Roche, Basel, Switzerland). The lysed cells were scraped and transferred to an Eppendorf tube for centrifugation at 500 × *g* for 5 min at 4°C. The supernatant corresponding to the cytosol and plasma membrane was removed and not assayed. The remaining pellet containing nuclei was washed with lysis buffer without NP-40, centrifuged at 500 × *g* for 5 min at 4°C, dissolved in 10 mM Tris–HCl, pH 7.4, with 2% SDS, sonicated, and stored at −20°C until further analysis. The total protein concentrations were measured using the Bio-Rad protein assay, according to the manufacturer’s instructions. Each cell fraction was diluted in Loading buffer (4xLDS, Ken-en-Tec, Taastrup, DK #B31010) and 0.05 M DTT (Sigma-Aldrich, St. Louis, MO, USA; #D-9770-10), boiled for 2 min; protein equivalents of 20 µg per lane were separated on a 10% SDS-PAGE gel. After separation, the proteins were electro-transferred to a nitrocellulose membrane for Western blotting. The presence of proteins expressed from the WT-*CASR*-pcDNA3.1(+), mutant-*CASR*-pcDNA3.1(+), WT-*CASR*- and mutant-*CASR*-pcDNA3.1(+), and the mock-vector was detected by incubating the membranes 16–24 h at 4°C with 1 µg/mL anti-*CASR* rabbit polyclonal antibody raised against amino acids 111–210 within the N-terminal ECD of human *CASR* (Santa Cruz Biotechnology Inc., Santa Cruz, CA, USA; Cat.# sc-33821). Subsequently, the membrane was incubated with a horseradish peroxidase-conjugated goat anti-rabbit secondary antibody at a dilution of 1:5,000 for 1 h at room temperature. The receptor proteins were visualized using chemiluminescence with the ECL Advance Western Blotting Detection reagent (GE Healthcare). The chemiluminescence of the *CASR* protein was detected using the ImageQuant LAS-4000, Fujifilm (GE Healthcare).

### Data Analysis and Statistics

Data from the [Ca^2+^]_o_ dose–response experiments were fitted to the four parameter dose-response curve using GraphPad Prism ver.6.0 (GraphPad, San Diego, CA, USA) the following variable slope equation: *R* = *R*_min_ + (*R*_max_ − *R*_min_)/(1 + 10^((LogEC50-^*^X^*^)^ * Hill slope)) where *R* is the response, *X* is the logarithm of the [Ca^2+^]_o_ concentration, *R*_max_ is the maximal response, *R*_min_ is the minimal response, EC50 is the agonist concentration that produces a half maximal response, and Hill slope describes the steepness of the curve. Based on the individual fitted curves the mean and SD values were calculated. To determine whether any statistically significant differences in *E*_max_ values were present, data were transformed to percent of the WT-*CASR E*_max_ (WT being 100%). Normality of data was tested by Shapiro–Wilks normality test. For normally distributed data (EC50 and Hill slope) one-way analysis of variance was used. For non-normally distributed data (*E*_max_) Kruskal–Wallis test was applied. Equality of variance between groups was tested by Brown–Forsythe and Bartlett’s test. Appropriate *post hoc* tests (the parametric Tukey’s multiple comparisons test and the non-parametric Dunn’s multiple comparisons test) were applied. All statistical analyses were done using GraphPad Prism v. 6.0. The statistical significance of induced [Ca^2+^]_o_ effects on the mutant- and WT-*CASR* regarding Hill slope, efficacy and potency, was considered at *P* < 0.05.

## Results

### Inheritance of a Novel Activating Mutation

**Figure 1:**
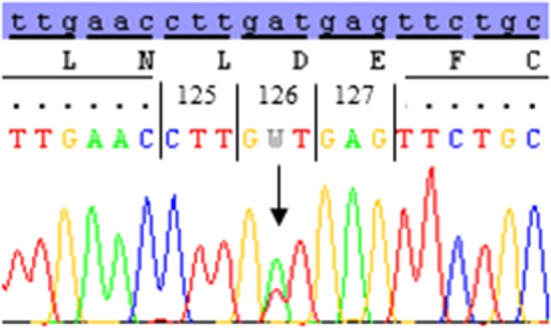
The DNA sequence of codon 123–129 of the calcium-sensing receptor (*CASR*) gene in the affected family members. The DNA sequence analysis of exon 3 reveal a nucleotide substitution of adenine (A) to thymidine (T) in codon 126 of the *CASR* gene; subsequently changing the amino acid aspartic acid (D) (GAT) to valine (V) (GTT). The heterozygous A/T mutant sequence of codon 126 was identified in I-2, II-2, and II-3.

A novel heterozygous genotype, D126V, of the *CASR* gene was identified, and the pedigree showed autosomal-dominant inheritance. The DNA sequence analysis revealed a novel missense mutation in exon 3 that was initiated by an adenine (A) to thymidine (T) substitution in codon 126(GAT), changing an aspartic acid (D) to valine (V) D126V(GTT) (Figure 1).

**Figure 2:**
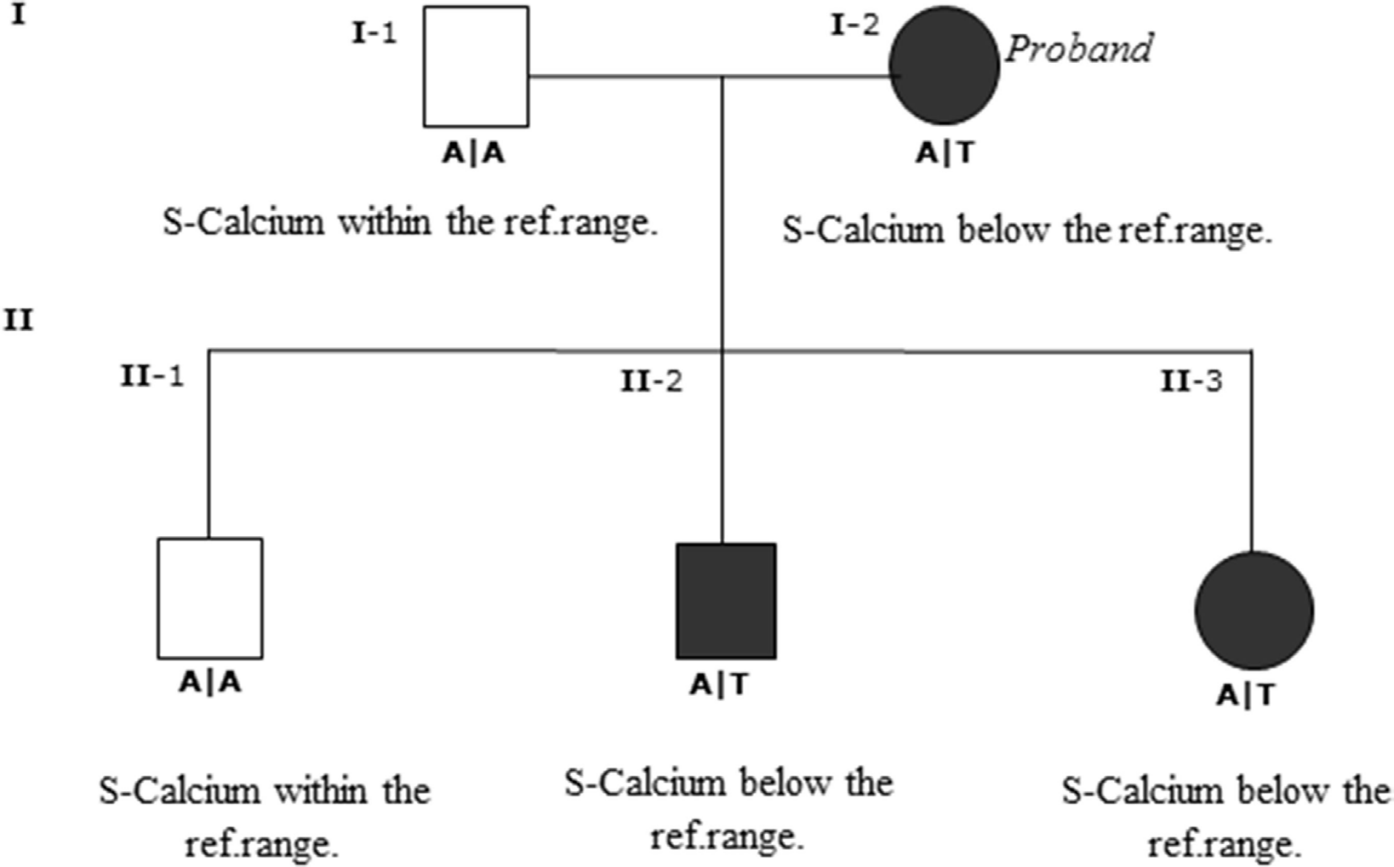
Genotype and pedigree of the family investigated. The nucleotide substitution was identified in I-2 (proband) and in II-2 and II-3 both affected progeny of the proband. The proband and the affected progeny all have S-calcium below reference range, I-2 had severe muscle pain, tetany, and depression; II-2 had gentle muscle pain; and II-3 had diffuse muscle pain and depression. Wild-type homozygous A/A of codon 126 is the open boxes; black boxes are the heterozygous mutant A/T codon.

The nucleotide substitution was identified in I-2 (proband), II-2, and II-3, all of whom were diagnosed with ADH, with S-calcium_(total)_ levels of 1.89–2.08 mmol/L (ref. 2.20–2.60 mmol/L) and S-iPTH levels ranging 0.6–1.09 pmol/L (ref. 1.1–6.9 pmol/L). The proband and two of her children were heterozygous carriers of the D126V mutation. The pedigree showed that D126V mutant allele was inherited in an autosomal-dominant manner (Figure 2), which is in accordance with the baseline biochemical parameters (Table 1).

### The Mutant *CASR*-Mediated IP Accumulation, Showing Increased Potency and a Decreased Hill Slope Compared to the WT Receptor

**Figure 3:**
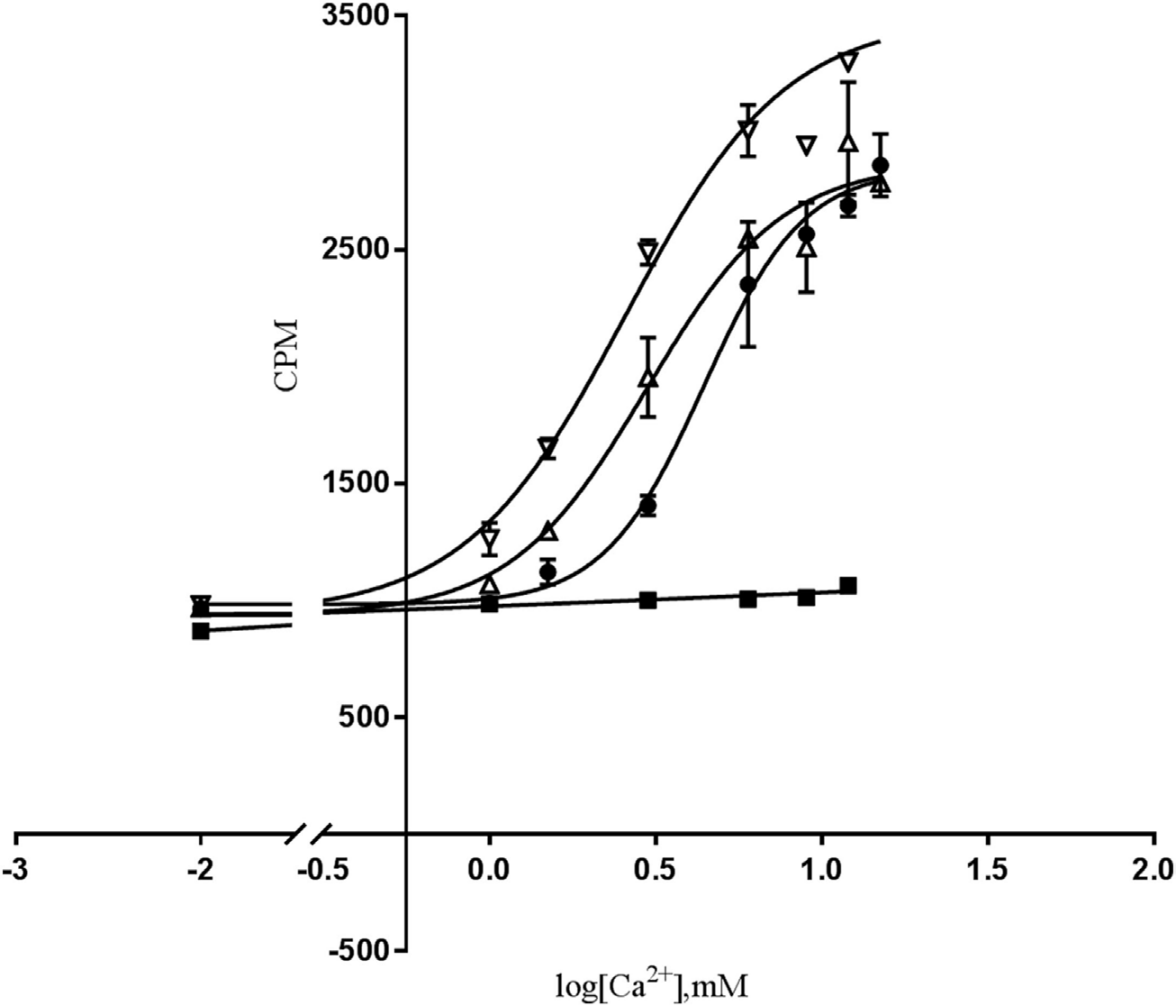
The effect of [Ca^2+^]_o_-induced PI-PLC activation in cells transfected with WT- or mutated calcium-sensing receptor (*CASR*) gene. Dose-response of IP accumulation (CPM) of [Ca^2+^]_o_-induced PI-PLC activation. Flp-IN human embryonic kidney-293 (HEK293) cells were transiently transfected with the wild-type calcium-sensing receptor (WT-*CASR*) (●), D126V-*CASR* (▿), WT/D126V-*CASR* (▵), or mock vector (■) were exposed to various [Ca^2+^]_o_ concentrations and IP accumulation was analyzed. Data are mean ± SD of a single representative experiment performed in duplicates. Four additional experiments gave similar results.

First, we determined the functional effects of the homozygous D126V-*CASR* and heterozygous D126V/WT-*CASR* receptors compared to the homozygous WT-*CASR* receptor at the first point of PI-PLC pathway activity *in vitro* by measuring IP accumulation in response to stimulation with 0.5–15 mM [Ca^2+^]_o_ for 60 min. We observed a more left-shifted response for cells expressing the homozygous D126V-*CASR* compared to the cells expressing heterozygous D126V/WT receptor and the cells expressing the WT-*CASR* (Figure 3).

Moreover, the potency increase was significant for the cells expressing D126V-*CASR* compared to the cells expressing the WT-*CASR* protein, the EC_50_ values were 3.24 ± 0.3 mM (*P* < 0.05) and 5.05 ± 0.7 mM, respectively, in accordance with a “gain-of-function” mutation. According to the D126V/WT-*CASR* there were no significant difference of the EC_50_ values compared to the WT-*CASR*, EC_50_ values were 3.57 ± 0.3 mM and 5.05 ± 0.7 mM, respectively.

The Hill slope of the homozygous D126V- and the heterozygous D126V/WT-*CASR* receptor was D126V = 2.22 ± 0.3, D126V/WT = 2.42 ± 0.1, than cells expressing the WT receptor revealed a Hill slope at 3.16 ± 0.4. The efficacy for both the D126V-*CASR* and the D126V/WT receptors were similar to the homozygous WT receptor, *E*_max_ = 111 ± 12% and 86 ± 9% (Table 2). The cells transfected with the mock vector did not show any dose-dependent response to [Ca^2+^]_o_ stimulation. The results for the homozygous genotype D126V-*CASR* are in accordance with a “gain-of-function" mutation of the CASR gene.

**Table 2:** Pharmacological parameters.

		IP acc.			(Ca^2**+**^)_i_-mobilization	
			
	EC_50_ mM	Hill slope	*E*_max_%	EC_50_ mM	Hill slope	*E*_max_%
WT-*CASR*	5.05 ± 0.7	3.16 ± 0.4	100	1.82 ± 0.2	1.96 ± 0.1	100
D126V-*CASR*	3.24 ± 0.3^a^	2.22 ± 0.3	111 ± 12	1.46 ± 0.2	2.15 ± 0.2	50 ± 4^b^
WT/D126V-*CASR*	3.57 ± 0.3	2.42 ± 0.1	86 ± 9	1.51 ± 0.2	2.15 ± 0.2	72 ± 4

*The potency (EC_50_), the Hill slope and the efficacy (*E*_max_) for [Ca^2+^]_o−_ stimulation of Flp-IN HEK293 cells transiently transfected with the WT-*CASR*, the D126V-*CASR*, the WT/D126V-*CASR*. Data shown as mean ± SEM from five/six experiments performed in duplicate*.

*^a^Significant from WT-CASR, P < 0.05*.

*^b^Significant from WT-CASR, P < 0.01*.

*WT-CASR, wild-type calcium-sensing receptor; HEK293, human embryonic kidney-293*.

### The Mutant *CASR*-Mediated (Ca^2+^)_i_ Mobilization, Showing Similar Hill Slope and Potency and Decreased Efficacy Compared to the WT Receptor

**Figure 4:**
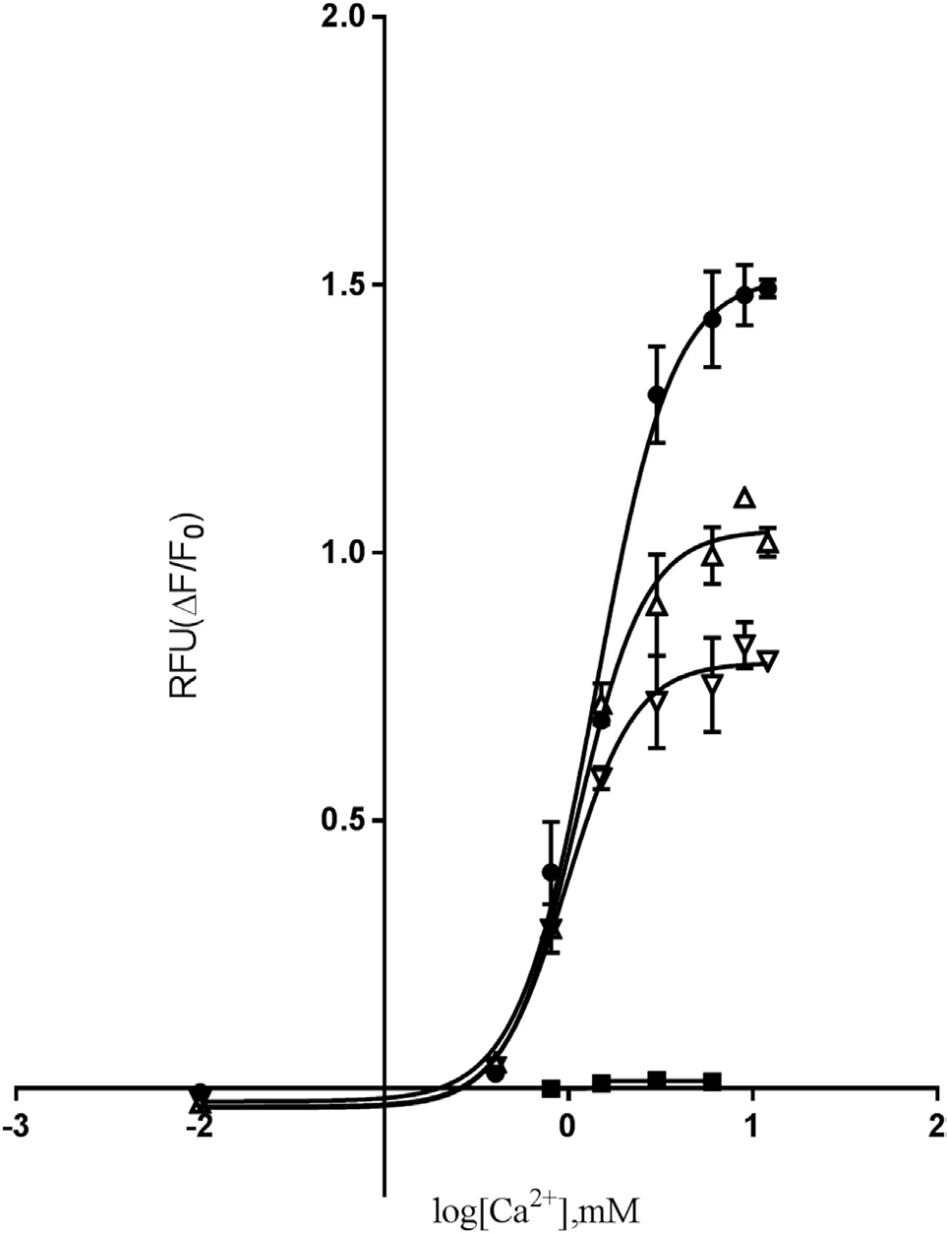
The effect of [Ca^2+^]_o_-induced (Ca^2+^)_i_-mobilization in cells transfected with WT- or mutated calcium-sensing receptor (*CASR*) gene. Dose-response of relative fluorescence units (RFU) of [Ca^2+^]_o_-induced (Ca^2+^)_i_-mobilization. Flp-IN human embryonic kidney-293 cells were transiently transfected with the wild-type calcium-sensing receptor (WT-*CASR*) (●), D126V-*CASR* (▿), WT/D126V-*CASR* (▵), or mock vector (■) were incubated with 4 µM fluo-4AM, washed, and (Ca^2+^)_i_ release was quantified after addition of various concentrations of [Ca^2+^]_o_. Data are mean ± SD of a single representative experiment performed in duplicates. Five additional experiments gave similar results.

We next investigated the effects of the mutation on downstream G_q/11_ signaling by measuring (Ca^2+^)_i_ mobilization in response to [Ca^2+^]_o_ stimulation. In the functional assay, FLIPR, Fluo-4 AM-treated cells were stimulated with 0.5–12 mM [Ca^2+^]_o_ for 60 s, and (Ca^2+^)_i_ mobilization was measured continuously. Interestingly, a remarkable reduction in the efficacy of the cells expressing the homozygous D126V-*CASR* was observed, as there was a highly significant decrease in the efficacy (E_max_) to 50 ± 10% (*P* < 0.01) compared to WT. For the cells expressing the heterozygous mutant *CASR* (WT/D126V) the efficacy was 72 ± 9% (Figure 4). By contrast, no differences in potency were observed between cells expressing the homozygous or heterozygous D126V mutations compared to the WT-*CASR*, with EC_50_ values of 1.46 ± 0.2 mM for D126V, 1.51 ± 0.2 mM for D126V/WT, and 1.82 ± 0.2 mM for WT (Table 2) (Figure 4).

Moreover, the Hill slope of the mutant receptors was not significantly different from the Hill slope in cells expressing the WT receptor, with Hill slopes for the homozygous D126V receptor of 2.15 ± 0.2, the heterozygous D126V/WT receptors of 2.15 ± 0.2, and the WT receptor of 1.96 ± 0.1 (Table 2).

### The Total Receptor Expression of the Mutant *CASR* Was Similar to the WT Receptor in the Immunoblot Analysis

**Figure 5:**
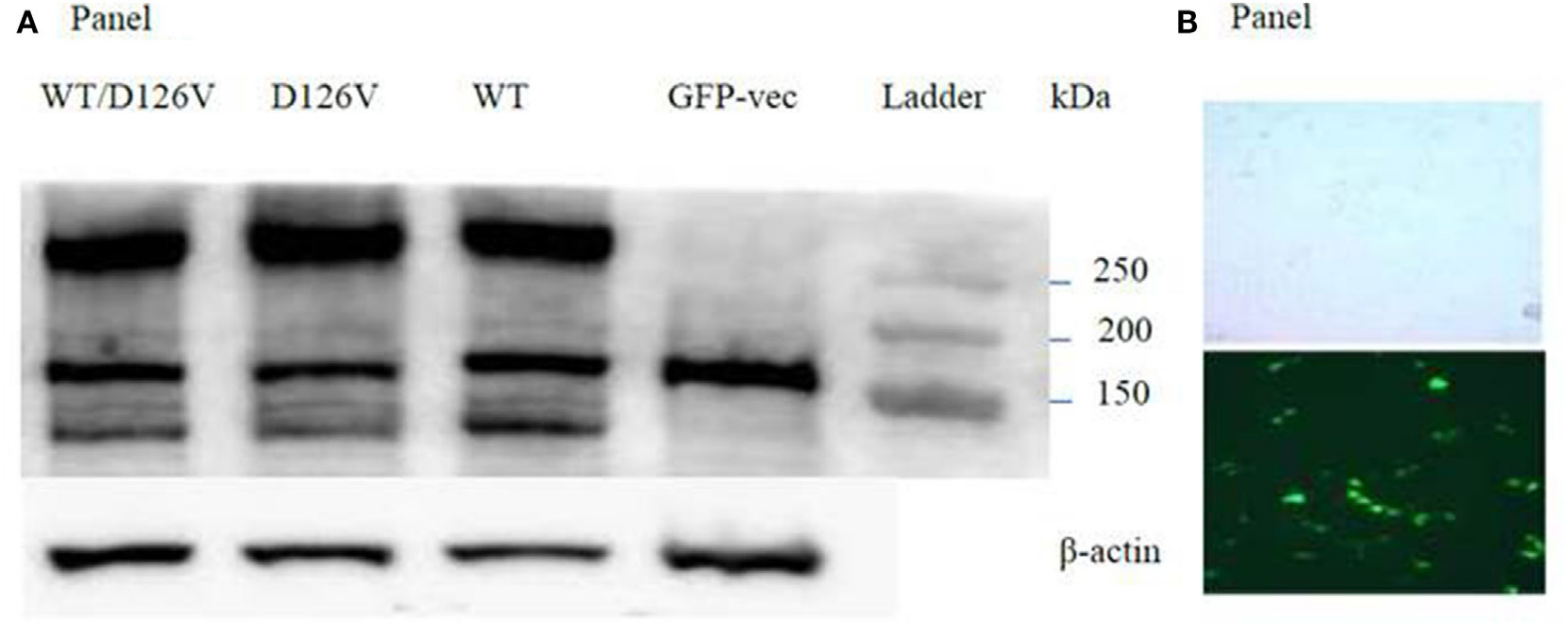
Evaluation of the WT- and the mutant calcium-sensing receptor (*CASR*) expression in transiently transfected Flp-In human embryonic kidney-293 (HEK293) cells. **(A)** Western blot analysis of the nuclear cell fraction, expression the band of the immature 140 kDa, the mature 160 kDa, and the functional dimeric receptor protein >240 kDa. Lane 1: WT/D126V-*CASR*, lane 2: D126V-*CASR*, lane 3: wild-type calcium-sensing receptor (WT-*CASR*), lane 4: mock-vector fused with GFP for transfections control, lane 5: ladder. WB on β-actin was used as loading control. Four additional experiments gave similar results. Panel **(B)** shows the light microscopy and the fluorescent microscopy of Fl-In HEK293 cells transfected with the GFP-vector.

Finally, we investigated the total receptor expression of the WT and mutant receptors. Western blot analysis of the nuclear cell fraction confirmed expression of the immature 140 kDa, the mature 160 kDa and the functional dimeric receptor protein >240 kDa (Figure 5A). The mature 160 kDa could not be assessed due to the presence of a band at the same size in the mock-vector (GFP) sample used as a transfection control. No gross differences were seen in D126V and the D126V/WT *CASR* dimeric >250 kDa band expression compared to the WT receptor, however, the D126V and D126V/WT CASR immature ~140 kDa species appear to have reduced expression compared to the WT receptor. This may indicate an abnormality in maturation of the receptor which might contribute to explaining the functional effects noted.

## Discussion

In this study, a novel, activating, missense mutation of the *CASR* gene, which is responsible for ADH, was identified and functionally characterized. The heterozygous GAT > GTT mutation in a family diagnosed with ADH was identified by analyzing the *CASR* sequence in exon 3, codon 126. The nucleotide substitution of an A to T resulted in a change from the amino acid aspartic acid (D) to valine (V). The genetic and clinical linkage of the proband and her children in the investigated family showed an autosomal-dominant inheritance pattern, as only two of her three children carry the allele with the D126V mutation. According to the summary of the baseline biochemical data shown in Table 1, I-2 and II-3 have hypocalcemic status, whereas the I-2 and II-2 have low PTH levels. Regarding the variable degree of ADH clinical phenotypes as seen in the affected family members, it is evident that I-2 and II-3 had symptoms of severe hypocalcemia, whereas II-2 had a form of asymptomatic mild hypocalcemia, which could indicate that the low S-calcium_(total)_ level together with the very low ionized calcium level might cause the severe clinical symptoms, as I-2 and II-3 had 1.9 and 2.1 mmol/L S-calcium_(total)_, respectively compared to 2.2 mmol/L S-calcium_(total)_, which is the borderline of the reference interval.

The novel D126V mutation described in this study is centrally located in the “Venus FlyTrap” domain and the hot-spot area known for gain-of-function mutations in the *CASR* gene. Seventeen different gain-of function mutations have been identified in eight codons (http://www.casrdb.mcgill.ca) in this functionally important region between amino acid 116 (alanine) and 131 (cysteine), indicating that this particular part of the receptor protein is sensitive to mutation-induced activation, consistent with the critical importance of this region in maintaining the inactive conformation of *CASR*, which was in accordance with the results of Jensen et al, among others (29). Furthermore, the D126V mutation identified in this study might alter the binding affinity and/or the dimerization of the receptor, as it is placed in this vital region that is highly important for the formation of the extracellular loop 2. This loop is involved in agonist-induced structural alterations and intermolecular disulfide linkages (31), and thus the formation of the *CASR* dimer (32), as described for the N124K mutation (33). In this study, we focused on the mechanism by which (Ca^2+^)_o_ activate two key points in the *CASR*-mediated PI-PLC signaling pathway by characterizing the functional consequences of this clinically relevant mutation. In contrast to other studies, we investigated the extent to which the heterozygosity had a pivotal impact on the functional effects of the *CASR*, which is very important because patients are heterozygous carriers of the mutant allele. Hence, we have identified mutation-induced impairments, including changes in agonist-stimulated signaling transduction *via* two distinct points of the PI-PLC pathway, IP accumulation and (Ca^2+^)_i_ mobilization, apparently without impaired receptor expression. Regarding the functional analysis of the dose-dependent response to extracellular calcium stimulation in the IP accumulation study, the homozygous D126V receptor was more sensitive to [Ca^2+^]_o_ compared to the WT receptor as it exhibited a significantly higher potency than the WT receptor. The D126V/WT receptor exhibited a better function due to the presence of one WT allele (Table 2; Figure 3). Surprisingly, the effects of the mutant D126V receptor on (Ca^2+^)_i_ mobilization were strikingly different than the effects on IP accumulation, in accordance with the study by Goolam et al. (27). [Ca^2+^]_o_ stimulation had little effect on (Ca^2+^)_i_ mobilization, as normalization of potency and Hill slope were observed for both the heterozygous and homozygous receptors. Conversely, a remarkably significant impaired efficacy of the cells expressing the homozygous D126V-*CASR* was shown for this later point of the G_q/11_ signaling pathway (Table 2; Figure 4), which is different from the results reported by Leach (26). We hypothesize that D126V enhances pathway desensitization by depleting the (Ca^2+^)_i_ stores and changing the intrinsic signaling capacity, rather than reducing the expression of the receptor. Indeed, the Western Blot analysis of the dimeric mutant receptor indicated no obvious differences from the WT receptor, though we cannot rule out that subtle differences in expression may have occurred that were not readily detectable by western blot (Figure 5). Unfortunately, we were not successful in specifically detecting the mature monomeric form of the receptor. However, we observed an apparent reduction in immature mutant receptor expression which may indicate a difference in the maturation of the mutant receptor protein and would be interesting to investigate in future studies. This study clearly showed that the naturally occurring mutation caused disturbances in ligand signaling, the results indicate that (Ca^2+^)_i_ mobilization by the PI-PLC pathway only is partially dependent on *CASR*-mediated activation, in accordance with Goolam et al. (27). The “gain-of-function” mutation in the *CASR* gene caused a significant increased potency at the first point of the PI-PLC signaling pathway, though in the later points downstream of the signaling pathway the D126V exhibited a significant reduced efficacy, however, the potency normalized. In the future, it will be of great interest to evaluate other pathways including G_i/o_ and G_12/13_ and the activity of intrinsic factors or different molecules, such as microRNA, that may contribute to stabilize the intercellular signaling in distinct types of cells (24, 25, 27, 34, 35). However, in future studies of *CASR* mutations cells endogenously expressing the receptor should be used as direction to assist in verifying clinical relevance of the mutation. Detailed knowledge of D126V will allow pharmacologists to design drugs that specifically target the molecular determinant that causes the impairments observed in ADH (26, 36). Overall, the present study confirms that the original model for GPCRs existing in either an “on” or “off” conformation is too simple, also in agreement with previous studies (25, 35, 37).

In conclusion, in this study, a family with a clinical diagnosis of ADH in two generations was evaluated to identify a mutation in the *CASR* gene and reveal an association between genotype and phenotype in the affected family members. The clinical condition was caused by a novel, activating, missense mutation (D126V) in the *CASR* gene and the *in vitro* functional characteristics of the mutation co-segregated with their individual phenotype.

## Ethics Statement

Informed consent was given from all participants. The study was approved by the Unit of Health Care, Regional Scientific Ethical Committee, The Capital Region of Denmark (J.no.17011317).

## Author Contributions

Idea of project AR and PS. Protocol, laboratory handling of material, and first draft: AR. Evaluation of results, final discussion, and writing of manuscript: AR, NJ, and PS. Statistics: AR and NJ.

## Conflict of Interest Statement

The authors declare that the research was conducted in the absence of any commercial or financial relationships that could be construed as a potential conflict of interest. The reviewer NK and handling Editor declared their shared affiliation.
