# Supplementary material for: Identification and Functional Characterization of a Novel Mutation in the Human Calcium-Sensing Receptor That Co-Segregates With Autosomal-Dominant Hypocalcemia
Source: Front Endocrinol (Lausanne). 2018 Apr 25;9:200. doi: 10.3389/fendo.2018.00200 (PMC5930847; doi:10.3389/fendo.2018.00200)
Supplement: Supplementary file 1 [file table_1.docx]

Supporting information

**Supplemental data:**

**S1 Table:** The nucleotide sequences for the Primers.

The nucleotide sequences of the forward and the reverse oligonucleotide primers for PCR and the sequence analysis of the *CASR* gene and the amplicon size (basepairs) of the PCR product.

| **Exon** | **Forward** | **Reverse** | **Amplicon size** |
| --- | --- | --- | --- |
| **1A** | **304 FW**  **GAGGTAGATGTTATCG CCATT** | **1208 RV**  **ACCCTCGGCACAGAGAACGTT** | **924** |
| **1B** | **966 FW**  **GCGCGCTGTGGAGT CGGGTAGA** | **1975 RV**  **AGCATTGGGCGTGAACGTGGG** | **1030** |
| **2** | **2 FW**  **CTGCAGGGAGTGAACTGCTCC** | **2 RV**  **ATGCTGAAGCTTATTGCCCCCAC** | **427** |
| **3** | **3 FW**  **GGGCTCTGTACAGAGCATGCC** | **3 RV**  **CCCAATAGCCATACGGTTTACCA** | **533** |
| **4** | **4 FW**  **GGATGCTCCCTCAAAGACTG** | **4 RV**  **GTGGTCATTTGAAAAGGGCA** | **1203** |
| **5** | **5 FW**  **CAG GGCACAGCCTACCTAAT** | **5 RV**  **CCTGAACTCTCTGGCCTTTG** | **437** |
| **6** | **6 FW**  **ACCAAGGACCTCTGGACCTC** | **6 RV**  **GTCAGTGAAGCCCATGGAAG** | **362** |
| **7A** | **7 FW A**  **ATGTCGGGGTTCAGCATATT** | **7 RV A**  **TGGATCTCCTTCATTCCAGC** | **913** |
| **7B** | **7 FW B**  **TGGTTTTCCTCTGCACCTT C** | **7 RV B**  **TCAGATTTGCTGTTCACCCA** | **1320** |
